# Adult height and all-cause and cause-specific mortality in the Japan Public Health Center-based Prospective Study (JPHC)

**DOI:** 10.1371/journal.pone.0197164

**Published:** 2018-05-14

**Authors:** Hikaru Ihira, Norie Sawada, Motoki Iwasaki, Taiki Yamaji, Atsushi Goto, Mitsuhiko Noda, Hiroyasu Iso, Shoichiro Tsugane, the JPHC Study Group

**Affiliations:** 1 Epidemiology and Prevention Group, Center for Public Health Sciences, National Cancer Center, Tokyo, Japan; 2 Department of Endocrinology and Diabetes, Saitama Medical University, Saitama, Japan; 3 Department of Public Health, Division of Social and Environmental Medicine, Osaka University, Graduate School of Medicine, Osaka, Japan; Public Health Agency of Canada, UNITED STATES

## Abstract

Adult height is determined by both genetic characteristics and environmental factors in early life. Although previous studies have suggested that adult height is associated with risk of mortality, comprehensive associations between height and all-cause and cause-specific mortality in the Japanese population are unclear. We aimed to evaluate the associations between adult height and all-cause and cause-specific mortality among Japanese men and women in a prospective cohort study. We investigated 107,794 participants (50,755 men and 57,039 women) aged 40 to 69 years who responded to the baseline questionnaire in the Japan Public Health Center-based Prospective Study. Participants were classified by quartile of adult height obtained from a self-reported questionnaire in men (<160cm, 160-163cm, 164-167cm, ≥168cm) and women (<149cm, 149-151cm, 152-155cm, ≥156cm). Hazard ratios (HR) and 95% confidence intervals (CI) for mortality from all-cause, cancer, heart disease, cerebrovascular disease, respiratory disease, and other cause mortality were calculated using Cox proportional hazards models. During follow-up, 12,320 men and 7,030 women died. Taller adult height was associated with decreased risk for mortality from cerebrovascular disease (HR _<160cm vs. ≥168cm_ (95% CI) = 0.83 (0.69–0.99); HR _for 5-cm increment_ (95% CI) = 0.95 (0.90–0.99)) and respiratory disease (HR _<160cm vs. ≥168cm_ (95% CI) = 0.84 (0.69–1.03); HR _for 5-cm increment_ (95% CI) = 0.92 (0.87–0.97)), but was also associated with increased risk for overall cancer mortality (HR _<160cm vs. ≥168cm_ (95% CI) = 1.17 (1.07–1.28); HR _for 5-cm increment_ (95% CI) = 1.04 (1.01–1.07)) in men. Taller adult height was also associated with decreased risk for mortality from cerebrovascular disease (HR _<149cm vs. ≥156cm_ (95% CI) = 0.84 (0.66–1.05); HR _for 5-cm increment_ (95% CI) = 0.92 (0.86–0.99)) in women. Our results confirmed that adult height is associated with cause-specific mortality in a Japanese population.

## Introduction

Among health-related factors, the intake of an adequate quality and amount of nutrition throughout infancy, childhood, and adolescence is crucially important to human growth and later health status [1,2]. Undernutrition in early life is well known to increase the risk of premature death [2]. Adult height is known to be determined by both genetic characteristics and environmental factors, including nutrition in early life [3], and has been used as an easily and widely available anthropometric marker. Previous epidemiological studies have generally suggested that taller adult height is associated with a decreased risk of mortality from stroke [4–9], cardiovascular disease [4,7–12], and respiratory disease [5–9], in contrast to increased risk of mortality from cancer [4–6,13–17]. Among potential reasons for this association of adult height with cardiovascular mortality, lower adult height has been associated with kidney malfunction and hypertension [18], and an increase in central aortic pressure [19]. With regard to cancer mortality, in contrast, a potential reason for the positive association between adult height and cancer mortality is explained by levels of insulin-like growth factor-1 (IGF-1) [20–23], which are related to the promotion of cell proliferation and inhibition of apoptosis [24].

A meta-analysis from a study of 1 million people reported that risk per 6.5-cm increase in height were a 6% decrease in death from vascular causes and a 4% increase for death from cancer [14], regardless of country or race. However, few prospective studies have examined comprehensive associations between adult height and major cause mortality such as cancer, heart disease, cerebrovascular disease, and respiratory disease in a Japanese population [4]. Therefore, the association of height and comprehensive outcomes, including all-cause and cause-specific mortality, requires confirmation in a large cohort. Furthermore, environmental conditions that play a critical role in establishing adult height [25,26] different even among Asian population. Thus, it is necessary to accumulate evidence on these associations in the Japanese population.

The purpose of this prospective cohort study was to evaluate the association between baseline adult height and subsequent risk of all-cause and cause-specific mortality in Japanese men and women.

## Methods

### Study cohort

The Japan Public Health Center-based Prospective Study (JPHC study) was started in 1990 for Cohort I and in 1993 for Cohort II. The study design has been described in detail previously [27]. In this study, all study subjects were residents of Japanese nationality who lived in the study areas at baseline. The participants were recruited from five Public Health Center areas (Iwate, Akita, Nagano, Okinawa-Chubu, and Tokyo) for Cohort I, and from six areas (Ibaraki, Niigata, Kochi, Nagasaki, Okinawa-Miyako, and Osaka) for Cohort II. The study population was defined as all residents aged 40–59 years in Cohort I and 40–69 years in Cohort II at the start of the respective baseline survey. In the baseline survey, a self-administered questionnaire was distributed to all registered residents, who were asked to report their sociodemographic characteristics, personal medical history, smoking and drinking history, diet and so on. The participants were informed of the objectives of the study, and those who completed the survey questionnaire were regarded as consenting to participation. The study was approved by the Institutional Review Board of the National Cancer Center, Tokyo, Japan. We enrolled a total of 140,420 individuals (68,722 men and 71,698 women) in Cohorts I and II. We excluded participants with non-Japanese nationality (n = 51), incorrect birth date (n = 7), and duplicate registration (n = 10), or those who emigrated before commencement of the starting point (n = 207). Of these, 113,258 participants responded to the baseline questionnaire (response rate 80.8%) and were included in the present study.

### Assessment of height and other covariates

The self-reported questionnaire asked about current height (cm) and weight (kg) at baseline. Body mass index (BMI) was calculated as weight (kg) divided by the height in meters squared (m^2^). Spearman’s correlation coefficients between self-reported height and weight, and measured height and weight were 0.94 and 0.95 in men, and 0.93 and 0.95 in women from our unpublished data, respectively. Adult height was divided into quartiles in men (<160cm, 160cm-163cm, 164cm-167cm, ≥168cm) and women (<149cm, 149cm -151cm, 152cm-155cm, ≥156cm). The questionnaire also included information on lifestyle factors and medical history, such as smoking status, alcohol consumption, leisure-time sports or physical exercise, history of hypertension, history of diabetes, menopausal status (women only) and age at menarche (women only), among others.

### Follow-up

We followed all registered cohort subjects from the starting point until 31 December 2013, except for the Tokyo and Osaka PHC areas, where follow-up was until the end of 2009 and 2012, respectively. Residency registration and death registration are required by the Basic Residential Register Law and Family Registry Law, respectively. We confirmed information on cause of death by death certificates, with permission of the Ministry of Health, Labour and Welfare. Analysis of cause-specific mortality followed the International Classification of Diseases and Related Health Problems, Tenth Revision (ICD-10) [28]. The major endpoint of this study was mortality from all causes, cancer (C00 to C97), heart disease (I20 to I52), cerebrovascular disease (I60 to I69), respiratory disease (J10 to J18 and J40 to J47), and other cause. Moreover, as a subtype analysis, we also analysed height-related cancer deaths (colorectal cancer (C18 to C20), pancreas cancer (C25), breast cancer (C50), ovary cancer (C56), prostate cancer (C61) and kidney cancer (C64 to C65)), given reports from the World Cancer Research Fund/American Institute for Cancer Research (WCRF/AICR) that increased adult height is a convincing or probable increased risk factor for these cancers [29,30]. We also analysed deaths from myocardial infarction (I21), haemorrhagic stroke (I60-I62) and ischemic stroke (I63).

### Statistical analysis

Among 113,258 participants, we excluded participants with a past history of cancer, stroke, or myocardial infarction (n = 4,100), and those with missing information on adult height and weight (n = 1,364), leaving 107,794 participants (50,755 men and 57,039 women) for inclusion in the analysis. Among them, person-years of follow-up were calculated for each subject from the data of the starting point to the date of death or end of the study period (31 December 2013), whichever occurred first. Subjects who were lost to follow-up were censored on the last confirmed date of presence in the study area.

The hazard ratios (HRs) and 95% confidence intervals (CIs) for mortality from all-cause, cancer, heart disease, cerebrovascular disease, respiratory disease, and other cause were calculated in men and women using a Cox proportional hazards model by quartiles of height, with the lowest height category as the reference. We performed the Schoenfeld Residuals Test, and confirmed the validated proportional hazards assumption in the Cox model. Covariates used in the model were birth year (continuous), smoking status (never, former, or current), alcohol consumption (never drinker, occasional drinker, 1–149 g/week, 150–299 g/week or ≥ 300 g/week), BMI (<18.5, 18.5–24, 25–29 or ≥30), history of hypertension (yes or no), history of diabetes (yes or no), leisure-time sports or physical exercise (almost never, 1–3 times/month or 1–2 times/week, or 2–3 times/week or almost every day), menopausal status (premenopausal or postmenopausal) and age at menarche (< 15 years or ≥ 15 years). We also calculated the HRs and 95% CIs for mortality by subtype of cancer, heart disease and cerebrovascular disease. Furthermore, we performed additional analyses by stratification of birth decade (1920s-1930s and 1940s-1950s), smoking status (never, former, or current), alcohol consumption (never, < 150 g/week or ≥ 150 g/week), and BMI (< 25 or ≥ 25), and calculated the HRs and 95% CIs in men and women. Trends were assessed by assignment of the ordinal value. All *P* values were 2-sided, and statistical significance was determined at the *P* < 0.05 level. All statistical analyses were carried out using the SAS program (SAS Institute Inc., Cary, NC, USA).

## Results

In present study, mean adult height and standard deviation were 164.3 ± 6.3 cm for men and 152.1 ± 5.6 cm for women. Table 1 shows baseline characteristics according to height category in men and women. Taller adults tended to be younger and more sedentary, and to have a greater body weight, lower BMI, more smoking, greater alcohol consumption, less hypertension and less diabetes, and an earlier age at menarche and lower prevalence of postmenopause (women only) than smaller adults in both men and women.

**Table 1:** Baseline characteristics of participants according to height category in men and women.

**Men (n = 50,755)**	**Quartile of height**			
	**<160cm**	**160-163cm**	**164-167cm**	**≥168cm**
Participants	9,840	13,039	12,174	15,702
Age (years), mean ± SD	55.1 ± 7.5	52.1 ± 7.7	50.7 ± 7.6	48.1 ± 7.2
Birth year (%)				
1920s	35.7	29.2	20.4	14.6
1930s	27.3	28.8	23.2	20.7
1940s	12.6	24.5	25.8	37.1
1950s	4.7	16.8	22.9	55.6
Body weight (kg), mean ± SD	56.9 ± 7.1	61.4 ± 7.4	64.2 ± 7.7	68.7 ± 8.8
BMI, mean ± SD	23.6 ± 2.9	23.6 ± 2.8	23.5 ± 2.8	23.4 ± 2.9
Smoking status (%)				
Never	23.5	26.5	23.2	26.8
Former	19.7	26.1	24.7	29.5
Current	17.4	25.1	24.1	33.4
Alcohol consumption (%)				
Never	22.8	27.2	23.8	26.3
Occasional	19.6	27.5	24.4	28.5
1–149 g/week	17.0	25.1	23.9	34.1
150–299 g/week	18.2	25.2	24.5	32.1
≥300 g/week	18.1	24.7	24.0	33.2
Sports or physical exercise almost daily (%)	9.2	9.9	9.5	8.8
History of hypertension (%)	19.3	16.5	17.1	14.3
History of diabetes (%)	6.9	6.4	6.4	5.3
**Women (n = 57,039)**	**Quartile of height**			
	**<149cm**	**149-151cm**	**152-155cm**	**≥156cm**
Participants	14,027	11,988	15,836	15,188
Age (years), mean ± SD	54.7 ± 7.9	52.3 ± 7.8	50.6 ± 7.6	48.6 ± 7.3
Birth year (%)				
1920s	43.2	22.7	21.2	12.9
1930s	31.4	23.8	26.1	18.7
1940s	18.1	20.2	30.3	31.4
1950s	8.4	13.4	30.1	48.1
Body weight (kg), mean ± SD	50.3 ± 6.9	52.9 ± 7.0	54.8 ± 7.3	57.5 ± 7.8
BMI, mean ± SD	23.9 ± 3.3	23.5 ± 3.1	23.3 ± 3.1	22.8 ± 3.1
Smoking status (%)				
Never	25.3	21.3	27.7	25.7
Former	16.5	18.9	27.1	37.5
Current	17.8	17.9	28.4	35.9
Alcohol consumption (%)				
Never	27.1	21.8	27.1	24.0
Occasional	17.9	19.7	30.3	32.1
1–149 g/week	15.6	18.1	29.9	36.4
150–299 g/week	15.8	16.6	28.5	39.1
≥300 g/week	16.5	18.5	27.7	37.2
Sports or physical exercise almost daily (%)	9.8	8.8	8.7	8.3
History of hypertension (%)	19.3	16.5	17.1	14.3
History of diabetes (%)	3.6	2.8	2.6	2.2
Age at menarche (years), mean ± SD	15.2 ± 2.1	14.7 ± 2.0	14.4 ± 1.8	14.1 ± 1.7
Postmenopausal (%)	71.5	60.6	52.3	41.4

BMI = body mass index, SD = standard deviation

In men, during 970,601 person-years of follow-up (average follow-up period, 19.1 years), 12,320 deaths occurred, including 4,897 from cancer, 1,525 from heart disease, 1,133 from cerebrovascular disease, 938 from respiratory disease and 3,827 from other causes (Table 2). In men, adult height was not associated with all-cause mortality (multivariate adjusted HR _<160cm vs. ≥168cm_ (95%CI) = 1.00 (0.94–1.05)). Taller adult height was associated with increased risk of cancer mortality (multivariate adjusted HR _<160cm vs. ≥168cm_ (95%CI) = 1.17 (1.07–1.28)), but also with decreased risk of cerebrovascular disease mortality (multivariate adjusted HR _<160cm vs. ≥168cm_ (95%CI) = 0.83 (0.69–0.99)) (Table 2). Additionally, taller adult height was associated with decreased risk of mortality from respiratory disease (multivariate adjusted HR _for 5-cm increment_ (95% CI) = 0.92 (0.87–0.97)) and other causes (multivariate adjusted HR _for 5-cm increment_ (95% CI) = 0.97 (0.94–0.99)) in men, although the HR of highest quartile showed no statistical significance (Table 2). Further, subtype analysis revealed statistically significant associations between adult height in men and mortality from colorectal cancer (multivariate adjusted HR _<160cm vs. ≥168cm_ (95% CI) = 1.32 (1.00–1.74); multivariate adjusted HR _for 5-cm increment_ (95% CI) = 1.07 (0.99–1.16)), and haemorrhagic stroke (multivariate adjusted HR _<160cm vs. ≥168cm_ (95% CI) = 0.67 (0.51–0.88); multivariate adjusted HR _for 5-cm increment_ (95% CI) = 0.89 (0.82–0.96)) (Table 2).

**Table 2:** Hazard ratios for all-cause and cause-specific mortality according to height category in men.

Men		n	Quartile of height										*P* for trend	Per 5-cm increment		
			<160cm	160-163cm			164-167cm			≥168cm						
			HR	HR	95% CI		HR	95% CI		HR	95% CI			HR	95% CI	
Participants		50,755	9,840	13,039			12,174			15,702						
All-cause	Person-years	970,601	184,316	250,352			234,221			301,712						
	Number of cases	12,320	3,290	3,382			2,775			2,873						
	Multivariate model		1	0.98	0.93	1.03	0.96	0.91	1.01	1.00	0.94	1.05	0.74	0.99	0.97	1.00
Cancer	Number of cases	4,897	1,175	1,381			1,123			1,218						
	Multivariate model		1	**1.11**	**1.02**	**1.20**	1.08	0.99	1.18	**1.17**	**1.07**	**1.28**	**< 0.01**	**1.04**	**1.01**	**1.07**
Height-related cancer																
Colorectal	Number of case	505	126	134			113			132						
	Multivariate model		1	1.07	0.82	1.38	1.09	0.83	1.44	**1.32**	**1.00**	**1.74**	0.05	1.07	0.99	1.16
Pancreas	Number of cases	314	67	94			84			69						
	Multivariate model		1	1.16	0.84	1.61	1.14	0.81	1.61	0.90	0.62	1.30	0.53	0.97	0.88	1.07
Prostate	Number of cases	188	59	64			34			31						
	Multivariate model		1	1.17	0.80	1.71	0.81	0.52	1.28	0.85	0.53	1.37	0.27	0.99	0.87	1.12
Kidney	Number of cases	75	16	23			17			19						
	Multivariate model		1	1.20	0.61	2.35	1.08	0.52	2.21	1.08	0.51	2.25	0.96	1.04	0.85	1.27
Heart disease	Number of cases	1,525	456	386			360			323						
	Multivariate model		1	**0.85**	**0.74**	**0.98**	0.92	0.79	1.07	0.86	0.73	1.01	0.13	0.96	0.92	1.00
Myocardial infarction	Number of cases	579	159	150			142			128						
	Multivariate model		1	0.91	0.72	1.15	0.97	0.76	1.24	0.96	0.74	1.24	0.87	0.99	0.92	1.06
Cerebrovascular disease	Number of cases	1,133	327	308			269			229						
	Multivariate model		1	0.88	0.75	1.04	0.94	0.79	1.12	**0.83**	**0.69**	**0.99**	0.10	**0.95**	**0.90**	**0.99**
Haemorrhagic stroke	Number of cases	512	140	144			121			107						
	Multivariate model		1	0.86	0.68	1.10	0.82	0.64	1.06	**0.67**	**0.51**	**0.88**	**< 0.01**	**0.89**	**0.82**	**0.96**
Ischaemic stroke	Number of cases	290	84	73			77			56						
	Multivariate model		1	0.90	0.64	1.27	1.28	0.92	1.79	1.01	0.69	1.47	0.45	1.01	0.92	1.12
Respiratory disease	Number of cases	938	341	245			175			177						
	Multivariate model		1	**0.76**	**0.64**	**0.91**	**0.71**	**0.59**	**0.86**	0.84	0.69	1.03	**0.02**	**0.92**	**0.87**	**0.97**
Other cause	Number of cases	3,827	991	1,062			848			926						
	Multivariate model		1	0.98	0.89	1.07	0.92	0.83	1.01	0.96	0.87	1.06	0.25	**0.97**	**0.94**	**0.99**

Multivariate model: adjusted for public health center, birth year (continuous), body mass index (<18.5, 18.5–24, 25–29 or ≥30), smoking status (never, former, or <20, 20–39, or ≥40 pack-years), alcohol consumption (never drinkers, occasional drinkers, 1–149 g/week, 150–299 g/week or ≥ 300 g/week), history of hypertension (yes or no), history of diabetes (yes or no) and leisure-time sports or physical exercise (almost never, 1–3 times/month or 1–2 times/week, or 2–3 times/week or almost every day).

In women, during 1,151,858 person-years of follow-up (average follow-up period, 20.2 years), 7,030 deaths occurred, including 2,630 from cancer, 920 from heart disease, 750 from cerebrovascular disease, 387 from respiratory disease and 2,343 from other causes (Table 3). In women, adult height was not associated with all-cause mortality (multivariate adjusted HR _<149cm vs. ≥156cm_ (95%CI) = 0.98 (0.91–1.05)) (Table 3). Taller adult height was associated with decreased risk of cerebrovascular disease mortality (multivariate adjusted HR _for 5-cm increment_ (95% CI) = 0.92 (0.86–0.99)) in women, although the HR of highest quartile showed no statistical significance (Table 3), and was not associated with mortality from cancer, heart disease, respiratory disease or other causes. Although we observed no statistically significant association between overall cancer mortality and adult height in women, mortality from ovary cancer (multivariate adjusted HRs _<149cm vs. ≥156cm_ (95% CI) = 2.22 (1.14–4.32); multivariate adjusted HR _for 5-cm increment_ (95% CI) = 1.34 (1.10–1.63)) was positively associated with adult height in women (Table 3). Additionally, adult height showed a non-statistically significant positive association with breast cancer mortality in women.

**Table 3:** Hazard ratios for all-cause and cause-specific mortality according to height category in women.

Women		n	Quartile of height										*P* for trend	Per 5-cm increment		
			<149cm	149-151cm			152-155cm			≥156cm						
			HR	HR	95% CI		HR	95% CI		HR	95% CI			HR	95% CI	
Participants		57,039	14,027	11,988			15,836			15,188						
All-cause	Person-years	1,151,858	283,426	243,878			320,878			303,675						
	Number of cases	7,030	2,372	1,533			1,762			1,363						
	Multivariate model		1	0.93	0.87	1.00	0.97	0.90	1.03	0.98	0.91	1.05	0.57	0.98	0.95	1.00
Cancer	Number of cases	2,630	797	556			694			583						
	Multivariate model		1	0.95	0.84	1.06	1.03	0.93	1.15	1.07	0.95	1.20	0.18	1.02	0.98	1.06
Height-related cancer																
Colorectal	Number of cases	348	109	66			92			81						
	Multivariate model		1	0.91	0.66	1.27	1.06	0.78	1.44	1.16	0.84	1.61	0.31	1.04	0.93	1.15
Pancreas	Number of cases	274	90	51			76			57						
	Multivariate model		1	0.84	0.58	1.20	1.07	0.77	1.49	0.95	0.65	1.37	0.92	1.00	0.89	1.13
Breast	Number of cases	180	40	37			44			59						
	Multivariate model		1	1.01	0.62	1.63	1.01	0.64	1.59	1.45	0.93	2.26	0.10	1.13	0.98	1.31
Ovary	Number of cases	92	15	15			29			33						
	Multivariate model		1	1.03	0.47	2.23	1.80	0.93	3.47	**2.22**	**1.14**	**4.32**	**< 0.01**	**1.34**	**1.10**	**1.63**
Kidney	Number of cases	36	15	7			8			6						
	Multivariate model		1	0.61	0.23	1.62	0.60	0.23	1.54	0.63	0.23	1.74	0.30	0.91	0.65	1.27
Heart disease	Number of cases	920	341	205			222			152						
	Multivariate model		1	0.94	0.78	1.14	0.96	0.80	1.16	0.94	0.76	1.16	0.55	0.98	0.91	1.04
Myocardial infarction	Number of cases	291	123	60			66			42						
	Multivariate model		1	0.84	0.60	1.17	0.77	0.55	1.08	0.76	0.51	1.11	0.09	0.91	0.81	1.02
Cerebrovascular disease	Number of cases	750	278	156			188			128						
	Multivariate model		1	**0.80**	**0.65**	**0.99**	0.88	0.72	1.08	0.84	0.66	1.05	0.14	**0.92**	**0.86**	**0.99**
Haemorrhagic stroke	Number of cases	410	142	93			106			69						
	Multivariate model		1	0.91	0.69	1.21	0.88	0.67	1.16	0.73	0.53	1.00	0.06	**0.87**	**0.76**	**0.99**
Ischaemic stroke	Number of cases	185	73	32			48			32						
	Multivariate model		1	0.68	0.43	1.07	1.05	0.71	1.55	1.06	0.67	1.66	0.64	1.02	0.89	1.17
Respiratory disease	Number of cases	387	157	88			84			58						
	Multivariate model		1	0.89	0.67	1.18	0.84	0.62	1.12	0.95	0.68	1.31	0.47	0.92	0.84	1.02
Other cause	Number of cases	2,343	799	528			574			442						
	Multivariate model		1	0.97	0.86	1.09	0.95	0.85	1.07	0.95	0.83	1.08	0.37	0.96	0.93	1.00

Multivariate model: adjusted for public health center, birth year (continuous), body mass index (<18.5, 18.5–24, 25–29 or ≥30), smoking status (never, former, or <20, 20–39, or ≥40 pack-years), alcohol consumption (never drinkers, occasional drinkers, 1–149 g/week, 150–299 g/week or ≥ 300 g/week), history of hypertension (yes or no), history of diabetes (yes or no), leisure-time sports or physical exercise (almost never, 1–3 times/month or 1–2 times/week, or 2–3 times/week or almost every day), menopausal status (premenopausal or postmenopausal) and age at menarche (< 15 years or ≥ 15 years).

Furthermore, on stratification of subjects by birth decade (1920s-1930s and 1940s-1950s), we observed a stronger inverse association between height and mortality from heart disease and cerebrovascular disease in younger subjects, with multivariate adjusted HRs (95%CI) of the highest versus lowest categories in the 1920s-1930s vs. 1940s-1950s of 0.87 (0.72–1.04) vs. 0.73 (0.54–0.98) for heart disease mortality and 0.86 (0.70–1.06) vs. 0.59 (0.42–0.84) for cerebrovascular disease mortality, respectively, in men (Table 4). In contrast, the association between adult height and mortality in women in the 1920s-1930s and 1940s-1950s were similar (S1 Table). Additionally, no substantial difference in results was seen on stratification by smoking status, alcohol consumption, BMI or age group (data not shown).

**Table 4:** Hazard ratios for all-cause and cause-specific mortality according to height category by birth decade in men.

**Men (birth decade in 1920s-1930s)**		**n**	**Quartile of height**										***P* for trend**	**Per 5-cm increment**		
			**<158cm**	**158-161cm**			**162-165cm**			**≥166cm**						
			**HR**	**HR**	**95% CI**		**HR**	**95% CI**		**HR**	**95% CI**			**HR**	**95% CI**	
Participants		23,484	4,845	5,449			6,995			6,195						
All-cause	Person-years	431,321	87,585	100,331			129,299			114,106						
	Number of cases	9,108	2,044	2,160			2,629			2,275						
	Multivariate model		1	0.97	0.91	1.04	0.96	0.90	1.02	1.00	0.94	1.07	0.96	1.00	0.98	1.02
Cancer	Number of cases	3,652	719	874			1,099			960						
	Multivariate model		1	**1.11**	**1.00**	**1.23**	**1.12**	**1.01**	**1.24**	**1.16**	**1.05**	**1.29**	**< 0.01**	**1.04**	**1.01**	**1.07**
Heart disease	Number of cases	1,135	292	240			338			265						
	Multivariate model		1	**0.79**	**0.66**	**0.95**	0.92	0.78	1.09	0.87	0.72	1.04	0.38	0.98	0.93	1.03
Cerebrovascular disease	Number of cases	861	207	202			255			197						
	Multivariate model		1	0.89	0.72	1.09	0.92	0.76	1.12	0.86	0.70	1.06	0.24	0.98	0.92	1.04
Respiratory disease	Number of cases	833	232	211			202			188						
	Multivariate model		1	0.88	0.72	1.07	**0.69**	**0.56**	**0.84**	0.82	0.67	1.01	**< 0.01**	**0.91**	**0.86**	**0.96**
Other cause	Number of cases	2,627	594	633			735			665						
	Multivariate model		1	0.96	0.86	1.08	0.90	0.80	1.01	0.99	0.88	1.12	0.61	0.99	0.95	1.02
**Men (birth decade in 1940s-1950s)**		**n**	**Quartile of height**										***P* for trend**	**Per 5-cm increment**		
			**<162cm**	**162-165cm**			**166-169cm**			**≥170cm**						
			**HR**	**HR**	**95% CI**		**HR**	**95% CI**		**HR**	**95% CI**			**HR**	**95% CI**	
Participants		27,271	5,875	7,625			5,713			8,058						
All-cause	Person-years	539,280	117,159	152,247			112,764			157,110						
	Number of cases	3,212	840	902			623			847						
	Multivariate model		1	**0.88**	**0.79**	**0.97**	**0.87**	**0.78**	**0.97**	**0.87**	**0.79**	**0.97**	**0.02**	**0.95**	**0.92**	**0.99**
Cancer	Number of cases	1,245	277	357			256			355						
	Multivariate model		1	1.04	0.89	1.23	1.11	0.93	1.32	1.13	0.95	1.33	0.13	1.05	0.99	1.10
Heart disease	Number of cases	390	112	113			74			91						
	Multivariate model		1	0.82	0.62	1.07	0.78	0.57	1.06	**0.73**	**0.54**	**0.98**	**0.04**	**0.88**	**0.80**	**0.96**
Cerebrovascular disease	Number of cases	272	88	78			47			59						
	Multivariate model		1	**0.72**	**0.52**	**0.98**	**0.61**	**0.42**	**0.88**	**0.59**	**0.42**	**0.84**	**< 0.01**	**0.83**	**0.75**	**0.92**
Respiratory disease	Number of cases	105	34	23			21			27						
	Multivariate model		1	0.63	0.36	1.11	0.79	0.43	1.44	0.87	0.50	1.52	0.84	1.00	0.84	1.19
Other cause	Number of cases	1,200	329	331			225			315						
	Multivariate model		1	**0.81**	**0.69**	**0.95**	**0.77**	**0.65**	**0.92**	**0.79**	**0.66**	**0.93**	**< 0.01**	**0.91**	**0.87**	**0.96**

Multivariate model: adjusted for public health center, birth year (continuous), body mass index (<18.5, 18.5–24, 25–29 or ≥30), smoking status (never, former, or <20, 20–39, or ≥40 pack-years), alcohol consumption (never drinkers, occasional drinkers, 1–149 g/week, 150–299 g/week or ≥ 300 g/week), history of hypertension (yes or no), history of diabetes (yes or no) and leisure-time sports or physical exercise (almost never, 1–3 times/month or 1–2 times/week, or 2–3 times/week or almost every day).

## Discussion

In this prospective cohort study in a large Japanese population, we found that taller adult height was associated with a decreased risk of mortality from cerebrovascular disease in men and women, and respiratory disease in men only, but an increased risk for mortality from cancer in men only. These findings are consistent with the results of several previous studies in other countries. To our knowledge, this is the first prospective cohort study to comprehensively investigate associations between adult height and four major causes of mortality in a Japanese population.

In Asia, while previous studies from Korea demonstrated an inverse association between adult height and all-cause mortality [6,9,31], some studies reported a null association, including studies from China [13] and the whole Asia-Pacific regions [4]. Our result in a Japanese population may support reports from the Chinese [13] and whole Asia-Pacific regions [4]. Our result of a null association between adult height and all-cause mortality is plausible, given that adult height is positively associated with cancer mortality and inversely with cardiovascular mortality.

One potential reason for the positive association between adult height and cancer mortality is explained by IGF-1, which are related to the promotion of cell proliferation and inhabitation of apoptosis [24]. Several previous studies have shown that high levels of IGF-1 are positively associated with an increased risk of several cancers, including colorectal [20], lung [21], breast [22] and prostate [23]. Although IGF-1 expression is promoted by growth hormone, which plays an essential role in determining adult height with bone growth [32], a higher level of serum IGF-1 in taller adults than those of shorter adults [33] may lead to an increased risk for cancer mortality. As a second reason, organs in larger bodies are larger than those in smaller bodies, and larger organs have a higher risk of dividing stem cells undergoing transformation to malignancy, with progression to cancer [15,34].

Although a positive association between height and cancer mortality has been reported [13–15,17], no study has yet reported a statistically significant association between adult height and overall cancer mortality in women. In women, we observed a statistically significant positive association between adult height and mortality from ovary cancer. Further, taller women tended to have an increased mortality risk from breast cancer, although we showed a non-statistically significant association. However, we showed a null association between height and overall cancer mortality, contrary to previous studies from Western countries [14,17]. The proportions of breast cancer death (6.8%) and ovary cancer death (3.5%) in this study were smaller than those in Western populations (breast 18.2%, ovary 6.3% [14]; breast 18.7%, ovary 8.7% [17]). We consider that the lower proportions for these cancers in Japanese may have led to the null association between overall cancer mortality and adult height in women. Because risks in cancer prevention should be proposed in a comprehensive and multi-factorial manner, adding our present results to the current risk prediction models for cancer prevention may allow the development of a more validated model. Although adult height cannot be changed, our results may contribute to the early detection of certain cancers as a public health action.

With regard to cardiovascular disease, several prospective studies have reported inverse associations between adult height and mortality from cardiovascular disease in Asian [4,6,9] and Western [5,7,8] populations. Low socioeconomic conditions in childhood might be considered a possible mechanism for an increased risk of cardiovascular disease [35,36]. These environmental factors in childhood may be reflected in the physical feature of short stature and may lead to an increased future risk of mortality from cardiovascular disease. Moreover, IGF-1 levels are generally inversely related to cardiovascular disease risk [37–39], because IGF-1 improves cardiovascular function and myocardial apoptosis [40], and is related to vascular endothelial function [38,41]. Additionally, shorter adult height has been associated with a faster heart rate and increased augmentation of the primary systolic pulse, leading to increased central aortic pressure [19]. Accordingly, and similar to the interpretation of previous studies, our present findings confirm the generally inverse association between adult height and mortality from cardiovascular disease, especially cerebrovascular disease. Previous studies have shown that adult height is strongly inversely associated with mortality of haemorrhagic stroke but less strongly associated with mortality of ischaemic stroke in Asian [4,6,9] and Western populations [14]. Further, the finding of associations between adult height and the risk of cardiovascular disease in a Japanese population is also consistent with findings regarding trends for haemorrhagic and ischaemic stroke [18]. Although differences in the association of adult height with subtype of stroke were not clear, one partial explanation is that haemorrhagic stroke may be more likely influenced by socioeconomic circumstances in childhood and adulthood than ischaemic stroke [14,35]. This evidence is strengthened by our similar results for an association between adult height and mortality from cerebrovascular disease.

Furthermore, our data show a stronger inverse association between height and mortality from heart disease and cerebrovascular disease in younger men. We considered that younger men might be more strongly influenced by IGF-1, given that they have a higher average height. Another reason may be differences in the prevalence of past disease history between young and old generations. Our results demonstrated that shorter men and women had a more frequent history of hypertension in the young generation (12.1% and 9.6% in shortest category versus 10.6% and 6.8% in the highest category for men and women, respectively), but that such difference was not seen in the old generation. Although we could not clearly explain the difference between young and old generations, previous papers have reported that shorter adults have a higher prevalence of hypertension than taller adults, because adult height is associated with coronary vessel diameter [4,7], and restriction of fetal growth is associated with kidney function, such as decreased filtration resulting from fewer nephrons [42].

In our study, although the prevalence of current smoking was higher among taller than shorter adults, we showed an inverse association between adult height and mortality from respiratory disease, in line with previous studies [5–9]. In many previous studies, an inverse association between adult height and mortality from respiratory disease was explained by differences in forced expiratory volume according to height [7,43]. Given a similar inverse association between never smokers, former smokers and current smokers, forced expiratory volume might play a more important role in mortality from respiratory disease. Additionally, lung health is susceptible to exposures in childhood, which are accumulated across life [7,44]. Therefore, exposures such as smoking and air pollution in childhood might influence both shorter adult height and increased risk of mortality from respiratory disease.

Strengths of our study include its prospective design with a large sample size, high response rate (80.8%), and low rate of loss to follow-up (0.8%). Further, information on death was exhaustive, because we used formal death registration in Japan to identify the causes of death. In contrast, our study also had several limitations. First, although nutritional status, passive smoking, air pollution, or socioeconomic status in early life, could affect development of adult height in childhood, we could not investigate these factors. Unfortunately, we obtained information on socioeconomic status such as education from participants in Cohort I only. We therefore adjusted for education level using these Cohort I subjects only, but found no substantial difference in results. Second, although we measured and adjusted for potential confounding factors, confounding by unmeasured variables or residual confounding cannot be excluded.

In conclusion, we found that adult height was inversely associated with mortality from cerebrovascular disease, particularly for haemorrhagic stroke in men and women, and from respiratory disease in men only. In contrast, we also found that adult height was positively associated with cancer mortality in men only.

## Supporting information

S1 Table Hazard ratios for all-cause and cause-specific mortality according to height category by birth decade in women. (DOCX)

## Abbreviations

BMI: body mass index
CI: confidence interval
HR: hazard ratio
ICD: International Classification of Diseases
IGF: insulin-like growth factor
JPHC study: Japan Public Health Center-based Prospective study
WCRF/AICR: World Cancer Research Fund/American Institute of Cancer Research
