# Supplementary material for: Adult height and all-cause and cause-specific mortality in the Japan Public Health Center-based Prospective Study (JPHC)
Source: PLoS One. 2018 May 14;13(5):e0197164. doi: 10.1371/journal.pone.0197164 (PMC5951564; doi:10.1371/journal.pone.0197164)
Supplement: S1 Table — (DOCX) [file pone.0197164.s001.docx]

**S1 Table. Hazard ratios for all-cause and cause-specific mortality according to height category by birth decade in women.**

| **Women (birth decade in 1920s-1930s)** | | **n** | **Quartile of height** | | | | | | | | | | ***P* for trend** | **Per 5-cm increment** | | |
| --- | --- | --- | --- | --- | --- | --- | --- | --- | --- | --- | --- | --- | --- | --- | --- | --- |
|  |  |  | **<147cm** | **147-149cm** | | | **150-153cm** | | | **≥154cm** | | |  |  |  |  |
|  |  |  | **HR** | **HR** | **95% CI** | | **HR** | **95% CI** | | **HR** | **95% CI** | |  | **HR** | **95% CI** | |
| Participants |  | 26,869 | 5,543 | 5,270 |  |  | 8,669 |  |  | 7,387 |  |  |  |  |  |  |
| All-cause | Person-years | 539,773 | 109,866 | 106,093 |  |  | 175,017 |  |  | 148,797 |  |  |  |  |  |  |
|  | Number of cases | 5,408 | 1,317 | 1,015 |  |  | 1,695 |  |  | 1,381 |  |  |  |  |  |  |
|  | Multivariate model |  | 1 | **0.87** | **0.79** | **0.95** | 0.96 | 0.89 | 1.04 | 0.96 | 0.88 | 1.04 | 0.86 | 0.98 | 0.95 | 1.00 |
| Cancer | Number of cases | 1,820 | 389 | 359 |  |  | 589 |  |  | 483 |  |  |  |  |  |  |
|  | Multivariate model |  | 1 | 1.02 | 0.88 | 1.20 | 1.09 | 0.95 | 1.26 | 1.09 | 0.94 | 1.26 | 0.18 | 1.02 | 0.97 | 1.06 |
| Heart disease | Number of cases | 790 | 204 | 152 |  |  | 244 |  |  | 190 |  |  |  |  |  |  |
|  | Multivariate model |  | 1 | 0.80 | 0.63 | 1.01 | 0.92 | 0.75 | 1.13 | 0.91 | 0.73 | 1.13 | 0.63 | 0.98 | 0.91 | 1.05 |
| Cerebrovascular disease | Number of cases | 590 | 165 | 96 |  |  | 196 |  |  | 133 |  |  |  |  |  |  |
|  | Multivariate model |  | 1 | **0.69** | **0.53** | **0.90** | 0.86 | 0.69 | 1.08 | **0.73** | **0.57** | **0.94** | 0.06 | **0.92** | **0.85** | **0.99** |
| Respiratory disease | Number of cases | 362 | 106 | 66 |  |  | 103 |  |  | 87 |  |  |  |  |  |  |
|  | Multivariate model |  | 1 | 0.73 | 0.52 | 1.01 | 0.75 | 0.56 | 1.02 | 0.87 | 0.63 | 1.18 | 0.37 | 0.93 | 0.84 | 1.03 |
| Other cause | Number of cases | 1,846 | 453 | 342 |  |  | 563 |  |  | 488 |  |  |  |  |  |  |
|  | Multivariate model |  | 1 | 0.86 | 0.74 | 1.00 | 0.95 | 0.83 | 1.09 | 0.98 | 0.85 | 1.13 | 0.90 | 0.97 | 0.93 | 1.01 |
| **Women (birth decade in 1940s-1950s)** | | **n** | **Quartile of height** | | | | | | | | | | ***P* for trend** | **Per 5-cm increment** | | |
|  |  |  | **<150cm** | **150-152cm** | | | **153-156cm** | | | **≥157cm** | | |  |  |  |  |
|  |  |  | **HR** | **HR** | **95% CI** | | **HR** | **95% CI** | | **HR** | **95% CI** | |  | **HR** | **95% CI** | |
| Participants |  | 30,170 | 5,949 | 6,857 |  |  | 8,778 |  |  | 8,586 |  |  |  |  |  |  |
| All-cause | Person-years | 612,085 | 123,608 | 140,322 |  |  | 177,420 |  |  | 170,735 |  |  |  |  |  |  |
|  | Number of cases | 1,622 | 368 | 398 |  |  | 432 |  |  | 424 |  |  |  |  |  |  |
|  | Multivariate model |  | 1 | 1.02 | 0.88 | 1.18 | 0.94 | 0.81 | 1.08 | 1.03 | 0.88 | 1.19 | 0.98 | 0.99 | 0.94 | 1.04 |
| Cancer | Number of cases | 810 | 163 | 193 |  |  | 231 |  |  | 223 |  |  |  |  |  |  |
|  | Multivariate model |  | 1 | 1.07 | 0.86 | 1.33 | 1.07 | 0.87 | 1.32 | 1.16 | 0.93 | 1.44 | 0.21 | 1.03 | 0.96 | 1.10 |
| Heart disease | Number of cases | 130 | 33 | 28 |  |  | 35 |  |  | 34 |  |  |  |  |  |  |
|  | Multivariate model |  | 1 | 0.77 | 0.46 | 1.32 | 0.91 | 0.56 | 1.50 | 1.02 | 0.61 | 1.69 | 0.82 | 0.99 | 0.84 | 1.17 |
| Cerebrovascular disease | Number of cases | 160 | 43 | 39 |  |  | 44 |  |  | 34 |  |  |  |  |  |  |
|  | Multivariate model |  | 1 | 0.95 | 0.59 | 1.51 | 0.94 | 0.60 | 1.47 | 0.82 | 0.50 | 1.34 | 0.44 | 0.95 | 0.81 | 1.11 |
| Respiratory disease | Number of cases | 25 | 8 | 9 |  |  | 4 |  |  | 4 |  |  |  |  |  |  |
|  | Multivariate model |  | 1 | 1.42 | 0.49 | 4.15 | 0.47 | 0.12 | 1.91 | 0.71 | 0.19 | 2.66 | 0.31 | 0.78 | 0.52 | 1.17 |
| Other cause | Number of cases | 497 | 121 | 129 |  |  | 118 |  |  | 129 |  |  |  |  |  |  |
|  | Multivariate model |  | 1 | 1.02 | 0.79 | 1.33 | 0.78 | 0.60 | 1.03 | 0.93 | 0.71 | 1.22 | 0.26 | 0.97 | 0.88 | 1.06 |

Multivariate model: adjusted for public health center, birth year (continuous), body mass index (<18.5, 18.5-24, 25-29 or ≥30), smoking status (never, former, or <20, 20-39, or ≥40 pack-years), alcohol consumption (never drinkers, occasional drinkers, 1-149 g/week, 150-299 g/week or ≥ 300 g/week), history of hypertension (yes or no), history of diabetes (yes or no), leisure-time sports or physical exercise (almost never, 1–3 times/month or 1-2 times/week, or 2-3 times/week or almost every day), menopausal status (premenopausal or postmenopausal) and age at menarche (< 15 years or ≥ 15 years).
